# Editorial: Robotic Assisted Laparoscopic Surgery (RALS) in Pediatric Urology

**DOI:** 10.3389/fped.2020.00085

**Published:** 2020-03-11

**Authors:** Mohan S. Gundeti, Miguel A. Castellan

**Affiliations:** ^1^Pediatric Urology, Section of Urology, Department of Surgery, The University of Chicago Medicine, Comer Children's Hospital, Pritzker School of Medicine, Chicago, IL, United States; ^2^Department of Pediatric Urology, Nicklaus Children's Hospital, Miami, FL, United States; ^3^Joe Di Maggio Children's Hospital, Hollywood, FL, United States

**Keywords:** robotic surgery, pediatric urology, RALS (robot-assisted laparoscopic surgery), minimaly invasive surgery, pyeloplasty

Since its inception within pediatric urology in early 2000, robotic assistance has become more widely accepted due to its aid in reconstructive aspects. Numerous procedures have been performed, from pyeloplasty to complex augmentation cystoplasty and Catherizable channels with bladder neck procedures. After the initial period of learning curve and feasibility assessments, quality outcomes and comparative studies have since been published, and these include some prospective studies. The ongoing challenges of the cost effectiveness, wider availability, and miniaturization of the instruments is a work in progress, and these will hopefully prove to be a short-term issue.

This Research Topic is intended to highlight the evolution of robotic surgical technology and its application in Pediatric Urology. In this collection of articles published on this subject, we have a wide selection of studies that will contribute to the further progress of the field. This topic continues to be of growing interest among the pediatric urology community and, in this Editorial, we have reviewed 14 accepted and included papers.

Authors from Chicago, IL, USA (Andolfi et al.), described the implementation, at their institution, of a 5-day mini-fellowship in robotic urologic surgery in children, and this was with a mentor, preceptor, and a proctor. The goal was to assist practicing pediatric urologists in incorporating robotic surgery into their practice. Between 2012 and 2018, a total of 29 national and international pediatric surgeons and urologists underwent robotic surgery training. Authors reported that this intensive program enabled surgeons to successfully incorporate the robotic platform into their practice and to advance the complexity of minimally invasive procedures.

An article from Toronto, ON, Canada (Fernandez and Farhat), described a comprehensive analysis of Robot-Assisted Surgery Uptake in Pediatric Surgery. This article described the historical publication uptake of RAS in pediatric urology and other surgical disciplines using a bibliometric comparison of the most cited manuscripts. They showed how the production of literature on the topics of pediatric laparoscopic and robot-assisted surgery has grown rapidly. Authors concluded that future directives need to focus on increasing the amount of evidence to support the innovation and development of pediatric instruments.

With newer technology, the learning curve is an important aspect, and (Sahadev et al.) described some the excellent tips to overcome this for the purposes of robotic-assisted laparoscopic extravesical ureteric reimplantation (RALUR- EV). Based on the institutional experience, it has been demonstrated that the learning curve of RALUR can be shortened with specific modifications predicated on experience; with technical adaptations, clinically significant improvements in surgical outcomes may be expected. The salient tips described are useful in order for early adopters to optimize the outcomes. The role of VCUG in the demonstration of reflux resolution is mandatory until a large volume of cases, such as open surgery, have been performed.

A manuscript from New York, NY, USA (Kim et al.), reviewed the specific considerations that are necessary to safely perform robotic-assisted laparoscopic procedures in infants, including physiological changes associated with pneumoperitoneum in infants, positioning, trocar placement, and docking. Young infants warrant special consideration when carrying out robotic surgery, and this paper has described the technical factors unique to this patient population.

Authors from Puerto Rico, USA (Morales-López et al.), reviewed current concepts in pediatric robotic-assisted Pyeloplasty. In this article, they have described the technique as it relates to the different robotic platforms, reviewed the surgical experience, and compared its results to other surgical approaches. They have also discussed patient and parent satisfaction, cost and financial considerations, along with evaluating the future of robotic surgery in the treatment of UPJ obstruction.

A minimal invasive approach for stone management is challenging, and (Ballesteros et al.) have carried out an excellent review of stone management; it has a special role in circumstances where other minimal invasive approaches have failed, complex urinary tract calculi, failed prior procedures, or abnormal genitourinary anatomy. The use of flexible or rigid ureteroscopy and a laparoscopic ultrasonic probe aid in the location of stones. A robotic approach remains the first choice of treatment for concomitant renal stones and ureteropelvic junction obstruction.

Infantile robotic surgery has been a challenge due to the generic system and instruments of the current robotic surgical system. Kim reviewed the early outcomes. According to the literature, the advantages of the reduced hospital stay and cosmetics with at par outcomes have been demonstrated. The main application has been pyeloplasty and heminephrectomy.

A manuscript from Atlanta, GA, USA (Bilgutay and Kirsch), has described the advantages and challenges of using robotic surgery in reconstructive surgeries involving the ureter in pediatric patients. This article described different applications, including upper ureteral reconstruction (e.g., pyeloplasty, UPJ polypectomy, ureterocalycostomy, and high uretero-ureterostomy in duplex systems), mid-ureteral reconstruction (e.g., mid uretero-ureterostomy for stricture or polyp), and lower ureteral reconstruction (e.g., ureteral reimplantation and lower ureter-ureterostomy in duplex systems). The different robotic procedures have been described in detail.

The robotic approach is beneficial in pelvic organ surgery, and (Gargollo and White) have described its role in bladder neck surgery for incontinence. The placement of an artificial sphincter to sling and close has been described along with the feasibility and safety of expanding the range of RAL surgical candidates to include selected patients with urinary incontinence.

Subramaniam, from Leeds, UK, reviewed the robotic approach to creating continent catheterizable channels (CC). This article described his personal experience with the robotic approach (18 consecutive cases), and has reviewed the published literature with regards to potential benefits, status, and outcomes. He reported that a robotic approach to CC is feasible and safe and that it has excellent outcomes and minimum morbidity.

The application of robotic assistance within general pediatric surgery was incredible already in early 2000, but it later did not gain much popularity due to the obstacle of miniaturization. Navarrete Arellano and González have described their experience of its successful application in nearly 100 patients, including infants and children, in cases involving the hepatobiliary-gastrointestinal tract and oncological and thoracic abnormalities.

Authors from Dallas, TX, USA (Chen and Peters), reviewed the status and future directions of robotic-assisted surgery in Pediatric Urology. This manuscript described very well the advantages and limitations of the system. The authors have described very clearly some of the reasons why other applications of robot-assisted surgery have failed to be adapted into routine practice in pediatric patients. The authors have also emphasized the importance of an open communication between physicians and engineers to develop new technology that can broaden the applicability of robot-assisted surgical technology in children.

The worldwide adoption of robotic assistance has been limited for multiple reasons, and important factors include the financial aspect of purchase and maintenance. Like other developing countries, South America has been no exception. Moldes et al. performed a survey of pediatric urologist from Brazil, Chile, Uruguay, and Argentina and elaborated upon some of these constrains from their experience. The most striking aspect compared to the USA was the lack of financial coverage of the procedure by insurance companies. The number of robots or trained surgeons available is minimal compared to the large pediatric population needed.

The future is always promising! The passion of innovators to overcome and offer the best services to patients in need drives further innovation. Sheth and Koh reviewed new robotic platforms and determined that they should include improved haptic feedback systems, flexible scopes, easier maneuverability, and even adaptive machine learning concepts to bring robotic-assisted laparoscopic surgery to the next level. The combination of virtual reality technology and robotic surgery may lead to a new era of digital surgery that may, in future, include automated robotic surgery.

## Author Contributions

MG and MC conceived of the presented idea, read all the manuscript and developed the Editorial together. Both supervised the findings of this work and discussed the results and contributed to the final manuscript.

### Conflict of Interest

The authors declare that the research was conducted in the absence of any commercial or financial relationships that could be construed as a potential conflict of interest.

